# Trends in clinical characteristics and outcomes of Pre-ART care at a large HIV clinic in Nairobi, Kenya: a retrospective cohort study

**DOI:** 10.1186/s12981-016-0122-y

**Published:** 2016-11-14

**Authors:** Jared O. Mecha, Elizabeth N. Kubo, Lucy W. Nganga, Peter N. Muiruri, Lilian N. Njagi, Immaculate N. Mutisya, Justine J. Odionyi, Syokau C. Ilovi, Mary Wambui, Christopher Githu, Richard Ngethe, Elizabeth M. Obimbo, Zipporah W. Ngumi

**Affiliations:** 1 Department of Clinical Medicine and Therapeutics, University of Nairobi School of Medicine, Nairobi, Kenya; 2 The Palladium Group, Nairobi, Kenya; 3 Kenyatta National Hospital, Nairobi, Kenya; 4 Centres for Disease Control & Prevention, Nairobi, Kenya

**Keywords:** HIV, Pre-ART, Attrition, Loss to follow up, Predictors, Risk factors, Nairobi, Kenya

## Abstract

**Background:**

The success of antiretroviral therapy in resource-scarce settings is an illustration that complex healthcare interventions can be successfully delivered even in fragile health systems. Documenting the success factors in the scale-up of HIV care and treatment in resource constrained settings will enable health systems to prepare for changing population health needs. This study describes changing demographic and clinical characteristics of adult pre-ART cohorts, and identifies predictors of pre-ART attrition at a large urban HIV clinic in Nairobi, Kenya.

**Methods:**

We conducted a retrospective cohort analysis of data on HIV infected adults (≥15 years) enrolling in pre-ART care between January 2004 and September 2015. Attrition (loss to program) was defined as those who died or were lost to follow-up (having no contact with the facility for at least 6 months). We used Kaplan-Meier survival analysis to determine time to event for the different modes of transition, and Cox proportional hazards models to determine predictors of pre-ART attrition.

**Results:**

Over the 12 years of observation, there were increases in the proportions of young people (age 15 to 24 years); and patients presenting with early disease (by WHO clinical stage and higher median CD4 cell counts), p = 0.0001 for trend. Independent predictors of attrition included: aHR (95% CI): male gender 1.98 (1.69–2.33), p = 0.0001; age 20–24 years 1.80 (1.37–2.37), p = 0.0001), or 25–34 years 1.22 (1.01–1.47), p = 0.0364; marital status single 1.55 (1.29–1.86), p = 0.0001) or divorced 1.41(1.02–1.95), p = 0.0370; urban residency 1.83 (1.40–2.38), p = 0.0001; CD4 count of 0–100 cells/µl 1.63 (1.003–2.658), p = 0.0486 or CD4 count >500 cells/µl 2.14(1.46–3.14), p = 0.0001.

**Conclusions:**

In order to optimize the impact of HIV prevention, care and treatment in resource scarce settings, there is an urgent need to implement prevention and treatment interventions targeting young people and patients entering care with severe immunosuppression (CD4 cell counts <100 cells/µl). Additionally, care and treatment programmes should strengthen inter-facility referrals and linkages to improve care coordination and prevent leakages in the HIV care continuum.

## Background

The HIV prevention and care continuum is a valuable framework for assessing linkage to, and retention in care, antiretroviral therapy and viral suppression for people living with HIV infection. Examining care and treatment programs in SSA (Sub-Saharan Africa) has revealed significant leakages across this continuum. For instance, only 80% of people testing positive for HIV are successfully linked to care within 3 months of diagnosis [1]. Worryingly, retention in care prior to ART (Antiretroviral Therapy) initiation is lower (45–75%), compared to retention after starting ART [2–4].

Attrition from care during this phase has been linked to early morbidity and mortality in the ART phase [5]. The reasons for low retention in pre-ART care include transportation costs, distance to health care facilities, young age, male gender, unemployment, lower education levels, stigma and fear of disclosure of HIV status [6]. Improving HIV/AIDS care and treatment program outcomes is dependent on successful linkage of patients to pre-ART care and retention in care until ART initiation [7]. The current WHO (World Health Organisation) HIV treatment guidelines recommend ART initiation in all patients with HIV irrespective of clinical stage or CD4 cell count [8]. Consequently, although the proportion of people in pre-ART care will be markedly reduced and the duration of pre-ART care shortened significantly, implementation of the new guidelines will not entirely eliminate a pre-ART phase. Evaluating the changing characteristics of pre-ART cohorts and how these characteristics influence retention and care outcomes can offer insights on designing interventions to improve retention and engagement in care prior to initiation of ART. Entry into pre-ART may also be viewed as a surrogate of effectiveness of population level prevention interventions.

We analysed routinely collected longitudinal clinical data to describe changing demographic and clinical characteristics of pre-ART cohorts over a 12 year period at a large urban HIV clinic in Kenya. We further described how these characteristics influence pre-ART attrition.

## Methods

### Study site and population

The Kenyatta National Hospital Comprehensive Care Centre (KNH CCC) offers ambulatory HIV care and treatment services mainly to residents of Nairobi city and neighbouring urban and peri-urban settlements. Most of the funds for these services were provided by the United States President’s Emergency Plan for AIDS Relief (PEPFAR) through the University of Nairobi’s AIDS Care and Treatment Services (2003–2010) and the Centres of Excellence Project (2010–2016). Patients registered at the KNH CCC are usually referred from the on-site HIV testing services (Voluntary Counselling and Testing—VCT, and Provider Initiated Testing and Counselling—PITC) and as formal and informal (walk-in) referrals. Most services are provided by a multi-disciplinary team of primary care providers. Patients with complications are booked for review by specialists. Outpatient HIV care is offered at no cost to the patients.

The criteria for pre-ART care and the organization and range of services changed, in line with national guidelines and better understanding of HIV care and treatment, during the 12 year follow-up period under review. Briefly, care comprises of registration and initial evaluation to assess eligibility for ART (WHO clinical stage and CD4 cell count), targeted additional laboratory tests, group and individual counselling and treatment literacy, cotrimoxazole prophylaxis; symptom-based tuberculosis (TB) screening and isoniazid prophylaxis (since 2014); linkage to psychosocial support groups; and regular follow-up (every 3–6 months depending on closeness to the ART eligibility threshold).

### Data collection, management and analysis

#### Data collection

Prior to 2013, providers recorded patient health information using semi-structured clinical encounter forms. Through an extensive exercise of data abstraction and reconstruction using fully structured clinical encounter forms, trained data assistants transferred key health information into a customized electronic health records (EHR) system. After 2013, all patient encounter sessions were entered directly into the EHR system in a paperless operating environment.

Data is captured at the point of care by all service providers. The EHR system has a data mining functionality that enables routine data quality checks using pre-defined clinical and program indicators.

#### Study design, study participants and setting

We conducted a retrospective cohort analysis of prospectively collected data on HIV infected adults (≥15 years) enrolling at the HIV Clinic between January 1, 2004 and September 30, 2015. The data analysis date was October 1, 2015. Patient records were excluded if: (i) they were missing the main outcome or explanatory variables of interest such as gender, age or date of birth; (ii) the enrolment date was before January 1, 2004, or after September 30, 2015; and (iii) age less than 15 years at enrolment.

#### Outcome definition

The outcome variable of interest was mode of transition from pre-ART care into one of these mutually exclusive categories: (i) started ART (irrespective of ARV treatment outcome); (ii) lost to program [died or lost to follow-up (no contact with the facility for at least 6 months)]; (iii) transferred to another facility before initiation of ART; or (iv) remained in care at the end of the study period. The main explanatory variable was the year of entry into pre-ART care. Other covariates of interest were demographic and clinical characteristics at enrolment into pre-ART care.

#### Data extraction

We extracted data from IQCare, an open-source, browser-based, EHR system custom-designed for HIV care and treatment programs in resource limited settings. The EHR has a data mining functionality, IQTools, which facilitates data quality assurance and extraction for reporting, quality improvement and operations research. Extracted data were exported to Microsoft Access (Microsoft Corporation, California) for analysis in SAS version 9.4 (SAS Institute, Cary, North Carolina). Extracted variables included pre-ART outcomes, year of enrolment, demographic characteristics such as gender, age at care start, marital status and residency (urban/rural). Clinical characteristics included CD4 cell count, WHO clinical stage and opportunistic infections at enrolment into pre-ART care. Other variables of interest were patient source, and date of HIV diagnosis.

To ensure confidentiality, data were stripped of patient identifiers such as names, home address and telephone numbers. In addition, the data analysts did not have access to individual patient data in the EHR and had no way of linking extracted records to any individual patient. Patient serial numbers were, however maintained for ease of merging datasets from different sources.

#### Statistical analysis

The main outcome variable was mode of transition out of pre-ART care. These were: started ART, attrition (lost to follow-up or death) and transferred to another facility. Some patients had not yet transitioned out of pre-ART care by the time of these analyses.

Covariates included demographic and clinical characteristics that could potentially influence mode of transition from pre-ART care. These were measured at care entry. Descriptive analyses were performed for these characteristics. Categorical variables were summarized using proportions while continuous variables were summarized using means and standard deviations or medians and interquartile ranges (IQR). The Chi square test was used to compare proportions, Mann-Whitney U test to compare medians and T-test to compare means. All statistical tests were two-sided at an alpha (α) level of 0.05.

In the time to event analyses (Kaplan-Meier survival analysis), the event of interest was pre-ART attrition. Patients without the event of interest i.e. those who started ART or remained in pre-ART care were censored while patients who transferred to another facility (n = 46) were excluded from the time to event analysis. We used Cox proportional hazards models to determine effect of patient characteristics at care entry on pre-ART attrition. A category for missing data was created in all covariates and incorporated in the Cox proportional hazards models.

To test the global null hypothesis that all parameter estimates for the covariates included in the model are equal to zero, we used the Likelihood Ratio, Score and Wald’s Chi square tests. Unadjusted and adjusted hazard ratios with 95% confidence intervals and p-values were generated and used to determine the patient characteristics that were independently associated with pre-ART attrition. Wald confidence limits were used for all Cox univariate/multivariate analyses. Data were analysed using SAS software 9.2 (SAS Institute, Cary, NC).

## Results

**Fig. 1:**
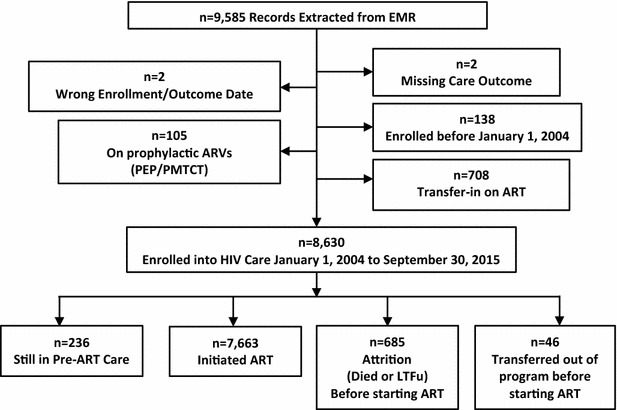
Flowchart of numbers

Between January 1, 2004 and September 30, 2015, 8630 adults were enrolled into HIV care, contributing a total of 88,126 patient-months of follow-up during 141 months of follow-up. At analysis, 7663 (88.8%) started ART, 236 (2.70%) remained in pre-ART care, 685 (7.9%) were lost to program, and 46 (0.5%) transferred to other providers (Fig. 1).

Table 1 shows patient enrolment characteristics stratified by pre-ART care start cohort. Overall, median age at enrolment was 37 years (IQR 31–44), and more than 60% of the patients were female. More than three quarters of the patients (78.2%) were urban residents. The on-site VCT centre was the main source of patients (43.4%). Over half of the patients (54.5%) were classified as WHO Clinical Stage I and II, and the median CD4 cell count at enrolment was 257 cells per cubic millimetre (IQR 109–460). Nearly a third (29.0%) of new enrolees had a CD4 count ≤200 cells per cubic millimetre. A tenth of patients had previous or current tuberculosis (TB) at enrolment.

**Table 1:** Enrolment characteristics by care start cohort

Year of enrolment	2004–05	2006–07	2008–09	2010–11	2012–13	2014–15	Overall	Chi Square for trend p value
	n = 789	n = 1435	n = 1538	n = 1438	n = 1830	n = 1600	n = 8630	
	n (%)	n (%)	n (%)	n (%)	n (%)	n (%)	n (%)	
*Gender*								
Female n (%)	493 (62.5)	920 (64.1)	995 (64.7)	914 (63.6)	1118 (61.1)	968 (60.5)	5408 (*62.7*)	0.0106
*Age (in yrs.)*								
Mean (sd)	38.2 (8.6)	38.3 (9.0)	37.6 (9.5)	37.3 (9.8)	37.3 (10.0)	36.8 (10.7)	*37.5 (9.7) *	
Median (IQR)	37 (32–43)	37 (32–44)	37 (31–43)	36 (30–43)	37 (30–44)	36 29–44)	*37 (31–44)*	
*Age group n (%)*								
15–19	3 (0.4)	8 (0.6)	21 (1.4)	19 (1.3)	44 (2.4)	45 (2.8)	140* (1.6)*	0.0001
20–24	14 (1.8)	46 (3.2)	66 (4.3)	93 (6.5)	118 (6.5)	143 (8.9)	480* (5.6)*	
25–34	275 (34.9)	478 (33.3)	528 (34.4)	506 (35.2)	583 (31.9)	514 (32.1)	2884* (33.4)*	
35–44	335 (42.5)	587 (40.9)	590 (38.3)	505 (35.1)	663 (36.2)	525 (32.8)	3205* (37.2)*	
45–54	121 (15.4)	239 (16.7)	254 (16.5)	233 (16.2)	328 (18)	282 (17.6)	1457* (16.9)*	
55+	41 (5.2)	77 (5.4)	79 (5.1)	82 (5.7)	94 (5.1)	91 (5.7)	464* (5.4)*	
*Marital status n (%)*								
Single	159 (20.2)	281 (19.6)	295 (19.2)	339 (23.6)	500 (27.3)	477 (29.8)	2051* (23.8)*	0.0034
Married	473 (60)	837 (58.3)	942 (61.3)	834 (58)	946 (51.7)	738 (46.1)	4770* (55.3)*	
Divorced/separated	38 (4.8)	105 (7.3)	93 (6.1)	90 (6.3)	169 (9.2)	171 (10.7)	666* (7.7)*	
Widowed	91 (11.5)	165 (11.5)	152 (9.9)	116 (8.1)	171 (9.3)	142 (8.9)	837* (9.7)*	
Not documented	28 (3.6)	47 (3.3)	56 (3.6)	59 (4.1)	44 (2.4)	72 (4.5)	306* (3.6)*	
*Residency n (%)*								
Rural	185 (23.5)	257 (17.9)	296 (19.3)	187 (13.0)	186 (10.2)	62 (3.9)	1173 (*13.6*)	0.0001
Urban	537 (68.1)	1081 (75.3)	1145 (74.5)	1182 (82.2)	1444 (78.9)	1362 (85.1)	6751* (78.2)*	
Not documented	67 (8.5)	97 (6.8)	97 (6.3)	69 (4.8)	200 (10.9)	176 (11.0)	706 (*8.2*)	
*Patient source n (%)*								
VCT	271 (34.4)	588 (41)	650 (42.3)	628(4 3.7)	793 (43.3)	812(5 0.8)	3742* (43.4)*	0.0001
PMTCT	202 (25.6)	296 (20.6)	293 (19.1)	232 (16.1)	159 (8.7)	107 (6.7)	1289* (14.9)*	
TB clinic	87 (11)	189 (13.2)	164 (10.7)	189 (13.1)	90 (4.9)	1 (0.1)	720* (8.3)*	
In-patient	59 (7.5)	114 (7.9)	129 (8.4)	98 (6.8)	174 (9.5)	190 (11.9)	764* (8.9)*	
Out-patient	9 (1.1)	16 (1.1)	14 (0.9)	22 (1.5)	63 (3.4)	11 (0.7)	135* (1.6)*	
Other facility	13 (1.7)	32 (2.2)	29 (1.9)	28 (2)	169 (9.2)	32 (2)	303* (3.5)*	
Other source	42 (5.3)	67 (4.7)	85 (5.5)	69 (4.8)	215 (11.8)	416(26)	894* (10.4)*	
Not documented	106 (13.4)	133 (9.3)	174 (11.3)	172 (12)	167 (9.1)	31 (1.9)	783* (9.1)*	
*HIV diagnosis n(%)*								
YoEnr ≠ YoDg	48 (6.1)	163 (11.4)	226 (14.7)	247 (17.2)	343 (18.7)	234 (14.6)	1261* (14.6)*	0.0412
YoEnr = YoDg	345 (43.7)	737 (51.4)	760 (49.4)	684 (47.6)	787 (43.0)	588 (36.8)	3901* (45.2)*	
YoDg not documented	396 (50.2)	535 (37.3)	552 (35.9)	507 (35.3)	700 (38.3)	778 (48.6)	3468* (40.2)*	
*Clinical stage n (%)*								
Stage 1&2	305 (38.7)	650 (45.3)	890 (57.9)	899 (62.5)	1043 (57)	915 (57.2)	4702* (54.5)*	0.0001
Stage 3&4	448 (56.8)	742 (51.7)	608 (39.5)	501 (34.8)	600 (32.8)	440 (27.5)	3339* (38.7)*	
Not documented	36 (4.6)	43 (3)	40 (2.6)	38 (2.6)	187 (10.2)	245 (15.3)	589* (6.8)*	
*CD4 count*								
Median (IQR)	178 (67–330)	197 (80–380)	273 (128–458)	320 (158–507)	279 (111–490)	259 (101–483)	*(257) (109–460)*	
*CD4 group n (%)*								
0–100	145 (18.4)	303 (21.1)	192 (12.5)	170 (11.8)	332 (18.1)	290 (18.1)	1432* (16.6)*	0.0001
101–200	86 (10.9)	214 (14.9)	166 (10.8)	182 (12.7)	232 (12.7)	189 (11.8)	1069* (12.4)*	
201–250	50 (6.3)	67 (4.7)	89 (5.8)	86 (6)	106 (5.8)	92 (5.8)	490* (5.7)*	
251–350	43 (5.5)	142 (9.9)	145 (9.4)	156 (10.9)	188 (10.3)	148 (9.3)	822* (9.5)*	
351–500	51 (6.5)	140 (9.8)	166 (10.8)	216 (15)	241 (13.2)	168 (10.5)	982* (11.4)*	
>500	47 (6)	159 (11.1)	194 (12.6)	288 (20)	341 (18.6)	285 (17.8)	1314* (15.2)*	
Not documented	367 (46.5)	410 (28.6)	586 (38.1)	340 (23.6)	390 (21.3)	428 (26.8)	2521* (29.2)*	
*Opportunistic infections n (%)*								
TB	91 (11.5)	178 (12.4)	161 (10.5)	144 (10)	231 (12.6)	133 (8.3)	938* (10.9)*	0.0726
PCP	33 (4.2)	60 (4.2)	73 (4.8)	74 (5.2)	89 (4.9)	26 (1.6)	355* (4.1)*	0.0271
Cryptococcal disease	4 (0.5)	8 (0.6)	16 (1)	9 (0.6)	15 (0.8)	9 (0.6)	61* (0.7)*	0.7077
Oral candidiasis	59 (7.5)	106 (7.4)	42 (2.7)	38 (2.6)	38 (2.1)	35 (2.2)	318* (3.7)*	0.0001
Oesophageal candidiasis	1 (0.1)	8 (0.6)	6 (0.4)	11 (0.8)	4 (0.2)	7 (0.4)	37* (0.4)*	0.9132
Kaposi’s sarcoma	12 (1.5)	12 (0.8)	20 (1.3)	12 (0.8)	8 (1)	11 (0.7)	85* (1)*	0.1126
Other OI^a^	197 (25)	423 (29.5)	441 (28.7)	502 (34.9)	648 (35.4)	402 (25.1)	2613* (30.3)*	0.0162
Any OI^b^	301 (38.2)	601 (41.9)	617 (40.1)	656 (45.6)	863 (47.2)	519 (32.4)	3557* (41.2)*	0.8563
*Presenting symptoms n (%)*								
Abdominal pain	38 (4.8)	53 (3.7)	55 (3.6)	57 (4)	76 (4.2)	85 (5.3)	364* (4.2)*	0.089
Headache	50 (6.3)	75 (5.2)	97 (6.3)	84 (5.8)	111 (6.1)	116 (7.3)	533* (6.2)*	0.21
Cough	167 (21.2)	206 (14.4)	264 (17.2)	217 (15.1)	315 (17.2)	284 (17.8)	1453* (16.8)*	0.8723
Chest pain	47 (6)	1 (2.9)	68 (4.4)	46 (3.2)	65 (3.6)	74 (4.6)	341* (4)*	0.6866
Any symptom	389 (49.3)	643 (44.8)	662 (43)	526 (36.6)	813 (44.4)	862 (53.9)	3895* (45.1)*	0.0369

Italic values indicate overall summary statistics

*VCT* voluntary counselling and testing; *PCP* pneumocystis carinii pneumonia

^a^Other OIs includes all major and minor OIs not listed in table* PMTCT* prevention of mother to child transmission

^b^Any OIs includes both OIs listed and not listed in table* TB* tuberculosis

*Time trends for enrolment characteristics* are presented in Table 1. There was a slight overall decline in the proportion of females newly entering care during the observation period (p = 0.0106 for trend). The proportion of adolescents and young adults (age 15–24 years) newly entering care increased progressively from 2.2% in 2004–05, to 11.7% in 2014–15 (p = 0.0001 for trend). The proportion of patients newly entering care from the on-site VCT progressively increased from 34.4% in 2004–05, to 50.8% in 2014–15 (p = 0.0001 for trend).

On average, 45% of patients were enrolled into care the same year they were diagnosed to be HIV positive. The proportion of patients enrolling in care in the same year that they were diagnosed to be HIV positive declined from 43.7% in 2004–05, to 36.8% in 2014–15 (p = 0.0412 for trend).

There was a progressive increase in the proportion of patients presenting early (in WHO Stage 1&2) for care (from 38.7% in 2004–05, to 57.2% in 2014–15, p = 0.0001 for trend). Similarly, there was an overall increase in median CD4 cell count at enrolment (from 178 to 259 cells/µl, p = 0.0001 for trend).

**Fig. 2:**
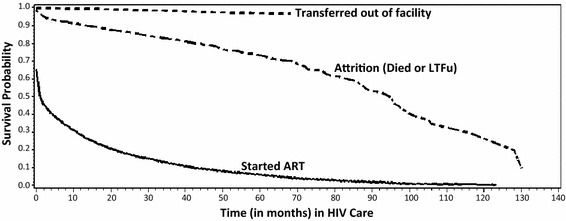
K-M curves plotted for transition from pre-ART care

Figure 2 shows the Kaplan-Meier curves for transition from pre-ART care. Median time to ART initiation was 2 months while median time to loss to program was 96 months.

Table 2 shows rates of attrition per 1000 patient-months of follow up for the different enrolment characteristics. More attrition was experienced by males compared to females, young adults (20–24 years) compared to those aged 35–44 years, single and non-documented marital status compared to married couples, urban and non-documented residency compared to rural, and patients who entered care with a high (>500 cells/µl) CD4 count compared to those with a CD4 count of 251–350 cells/µl. On the other hand, less attrition was noted among patients whose source of entry into care was the PMTCT or TB clinics compared to the on-site VCT, patients with TB at entry into care compared to those without TB. Additionally, patients with a documented opportunistic condition experienced less attrition compared to those without an opportunistic infection at entry into care.

**Table 2:** Attrition (/1000patient-months of follow-up) by enrolment characteristics

Enrolment characteristic	Numb dead or LTFu	Total months in care	Attrition *(/1000 patient*−*mths of follow*-*up)* (95% CI)
Overall	685	88,126	7.8 (7.2–8.4)
*Gender*			
Female	363	58,886	6.2 (5.5–6.8)
Male	322	29,240	11.0 (9.8–12.2)
*Age group*			
15–19	12	918	13.1 (5.7–20.4)
20–24	86	5191	16.6 (13.1–20.0)
25–34	295	36,680	8.0 (7.1–9.0)
35–44	209	29,531	7.1 (6.1–8.0)
45–54	68	12,454	5.5 (4.2–6.8)
55Plus	15	3352	4.5 (2.2–6.7)
*Marital status*			
Single	230	18,942	12.1 (10.6–13.7)
Married	335	52,154	6.4 (5.7–7.1)
Divorced	43	5393	8.0 (5.6–10.3)
Widowed	39	7934	4.9 (3.4–6.5)
Not documented	38	3703	10.3 (7.0–13.5)
*Residency*			
Rural	65	14,908	4.4 (3.3–5.4)
Urban	553	66,661	8.3 (7.6–9.0)
Not documented	67	6557	10.2 (7.8–12.7)
*Point of entry into care*			
VCT	327	38,075	8.6 (7.7–9.5)
PMTCT	66	17,440	3.8 (2.9–4.7)
TB Clinic	37	9731	3.8 (2.6–5.0)
Inpatient	46	6150	7.5 (5.3–9.6)
Outpatient	12	1061	11.3 (4.9–17.7)
Other facility	11	1049	10.5 (4.3–16.7)
Other source	63	4280	14.7 (11.1–18.3)
Not documented	123	10,340	11.9 (9.8–14.0)
*YoEnr vs YoDg*			
YoEnr ≠ YoDg	59	10,027	5.9 (4.4–7.4)
YoEnr = YoDg	257	45,336	5.7 (5.0–6.4)
YoDg not documented	369	32,763	11.3 (10.1–12.4)
*Disease stage (WHO)*			
Stage 1–2	354	62,334	5.7 (5.1–6.3)
Stage 3–4	119	24,099	4.9 (4.1–5.8)
Not documented	212	1693	125.2 (109.5–141.0)
*CD4 count*			
0–100	37	6218	6.0 (4.0–7.9)
101–200	28	5561	5.0 (3.2–6.9)
201–250	8	2917	2.7 (0.8–4.6)
251–350	31	9506	3.3 (2.1–4.4)
351–500	85	19,328	4.4 (3.5–5.3)
> 500	216	34,222	6.3 (5.5–7.2)
Not documented	280	10,374	27.0 (23.9–30.1)
*TB*			
No	654	80,707	8.1 (7.5–8.7)
Yes	31	7419	4.2 (2.7–5.6)
*PCP*			
No	662	83,205	8.0 (7.4–8.6)
Yes	23	4921	4.7 (2.8–6.6)
*Oral candidiasis*			
No	673	84,681	7.9 (7.3–8.5)
Yes	12	3445	3.5 (1.5–5.5)
*Other OI*			
No	504	51,738	9.7 (8.9–10.6)
Yes	181	36,388	5.0 (4.3–5.7)
*Any OI*			
No	472	44,453	10.6 (9.7–11.6)
Yes	213	43,673	4.9 (4.2–5.5)
*Cryptococcal disease*			
No	683	87,641	7.8 (7.2–8.4)
Yes	2	485	4.1 (0–9.8)
*Oesophageal candidiasis*			
No	679	87,725	7.7 (7.2–8.3)
Yes	6	401	15.0 (3.1–26.8)
*Kaposi’s sarcoma*			
No	681	87,567	7.8 (7.2–8.4)
Yes	4	559	7.2 (0.2–14.1)
*Lymphoma*			
No	684	88,084	7.8 (7.2–8.3)
Yes	1	42	23.8 (0–69.9)

*VCT* voluntary counselling and testing; *PMTCT* prevention of mother to child transmission; *TB* tuberculosis; *PCP* pneumocystis carinii pneumonia; *OI* opportunistic infection

Overall, risk factors for pre-ART attrition included: aHR (95% CI); male gender 1.98 (1.69–2.33), p = 0.0001 compared to female; age 20–24 years 1.80 (1.37–2.37), p = 0.0001, or 25–34 years 1.22 (1.01–1.47), p = 0.0364 compared to age 35–44 years; marital status single 1.55 (1.29–1.86), p = 0.0001 or divorced 1.41 (1.02–1.95), p = 0.0370 compared to married; urban residency 1.83 (1.40–2.38), p = 0.0001 compared to rural; CD4 count of 0–100 cells/µl 1.63 (1.003–2.658), p = 0.0486 or CD4 count >500 cells/µl 2.14 (1.46–3.14), p = 0.0001 compared to 251–350 cells/µl. Non-documentation of: marital status 1.53 (1.08–2.17), p = 0.0164; care entry point 1.52 (1.21–1.90), p = 0.0003; clinical stage 10.5 (8.03–13.6), p = 0.0001; and CD4 count 2.98 (2.00–4.44), p = 0.0001 also predicted attrition. On the other hand, factors which rendered attrition less likely included: aHR: (95% CI): patient referrals from the PMTCT clinic: 0.54 (0.41–0.71), p = 0.0001, and TB clinic: 0.55 (0.39–0.78), p = 0.0007 compared to on-site VCT (Table 3).

**Table 3:** Hazard ratios for pre-ART attrition

Unadjusted and adjusted hazard ratios for pre-ART attrition				
Enrolment characteristic	Unadjusted		Adjusted	
	HR (95% CI)	p value	HR (95% CI)	p value
*Gender*				
Female	Ref	Ref	Ref	Ref
Male	1.71 (1.47–1.99)	0.0001	*1.98 (1.69–2.33)*	*0.0001*
*Age*				
15–19	1.75 (0.98–3.13)	0.0596	1.39 (0.76–2.54)	0.2833
20–24	2.55 (1.99–3.28)	0.0001	*1.80 (1.37–2.37)*	*0.0001*
25–34	1.25 (1.05–1.50)	0.0133	*1.22 (1.01–1.47)*	*0.0364*
35–44	Ref	Ref	Ref	Ref
45–54	0.78 (0.59–1.02)	0.0683	0.77 (0.59–1.02)	0.0680
≥55	0.58 (0.34–0.98)	0.0416	0.68 (0.40–1.16)	0.1526
*Marital status*				
Single	1.81 (1.53–2.14)	0.0001	*1.55 (1.29–1.86)*	*0.0001*
Married	Ref	Ref	Ref	Ref
Divorced	1.13 (0.82–1.55)	0.4533	*1.41 (1.02–1.95)*	*0.0370*
Widowed	0.72 (0.52–1.00)	0.0511	1.02 (0.73–1.44)	0.8907
Not documented	1.68 (1.20–2.36)	0.0024	*1.53 (1.08–2.17)*	*0.0164*
*Residency*				
Rural	Ref	Ref	Ref	Ref
Urban	1.80 (1.39–2.32)	0.0001	*1.83 (1.40–2.38)*	*0.0001*
Not documented	2.18 (1.55–3.07)	0.0001	0.97 (0.68–1.39)	0.8573
*Care entry point*				
VCT	Ref	Ref	Ref	Ref
PMTCT	0.47 (0.36–0.61)	0.0001	*0.54 (0.41–0.71)*	*0.0001*
TB clinic	0.48 (0.34–0.67)	0.0001	*0.55 (0.39–0.78)*	*0.0007*
In patient	0.82 (0.60–1.11)	0.2018	1.02 (0.74–1.40)	0.9082
Other facility	0.82 (0.45–1.50)	0.526	1.00 (0.54–1.85)	0.9937
Other source^a^	1.32 (1.02–1.69)	0.0334	0.99 (0.76–1.28)	0.9177
Not documented	1.43 (1.16–1.76)	0.0008	*1.52 (1.21–1.90)*	*0.0003*
*HIV diagnosis to care entry*				
12 months or less	Ref	Ref	Ref	Ref
More than 12 months	1.18 (0.86–1.62)	0.3123	1.32 (0.96–1.83)	0.0916
Not documented	1.92 (1.64–2.24)	0.0001	1.03 (0.86–1.24)	0.7452
*Clinical stage*				
WHO stage 1&2	Ref	Ref	Ref	Ref
WHO stage 3&4	0.76 (0.62–0.94)	0.0119	0.83 (0.66–1.05)	0.1222
Not documented	14.5 (12.1–17.5)	0.0001	*10.5 (8.03–13.6)*	*0.0001*
*CD4 count*				
0–100	1.30 (0.81–2.10)	0.2802	*1.633 (1.003–2.658)*	*0.0486*
101–200	1.22 (0.73–2.04)	0.442	1.42 (0.85–2.37)	0.1856
201–250	0.69 (0.32–1.51)	0.3579	0.73 (0.34–1.60)	0.4358
251–350	Ref	Ref	Ref	Ref
351–500	1.45 (0.96–2.18)	0.0796	1.51 (1.00–2.29)	0.0506
>500	2.09 (1.43–3.05)	0.0001	*2.14 (1.46–3.14)*	*0.0001*
Not documented	5.86 (4.03–8.52)	0.0001	*2.98 (2.00–4.44)*	*0.0001*
*Opportunistic infections (OI) *				
Any OI	0.49 (0.41–0.57)	0.0001	0.91 (0.76–1.10)	0.3419

Italic values indicate statistically significant (p < 0.05) adjusted Hazard Ratios with corresponding 95% Confidence Intervals

^a^Other patient source includes out-patient, KNH CCC and others.* PMTCT* prevention of mother to child transmission

*VCT* voluntary counselling and testing; *TB* tuberculosis

## Discussion

We have described overall trends in pre-ART patient characteristics and predictors of attrition prior to initiation of ART over a 12 year period in a large urban clinic cohort in Kenya. At analysis, nearly 89% of patients enrolled into pre-ART care had started ART, and the overall loss to program was approximately 8%.

Consistent with other studies [9, 10], most of the patients newly enrolling into care were young females reflecting the disproportionate burden of HIV infection in this group. However, the extent of this disparity, as measured using care and treatment data, has come under scrutiny. In a recent innovative analysis, Auld et al [11] compared the female to male ratio among new ART enrolees with that of HIV infected adults in the general population. In this analysis of data from 12 countries, 17–73% fewer men (aged above 15 years) were enrolling in ART compared to females. Possibly, HIV infected men are not accessing care to the same extent as women. This may be due, in part, to lower emphasis on men’s health in media campaigns, fewer opportunities for males to be tested for HIV, and probably, greater perception of stigma and discrimination among men [12].

Worryingly, the proportion of adolescents and young adults (age 15–24 years) newly entering care increased five-fold during the observation period. Similar findings have been documented previously. A study conducted in Kenya showed an increase in the number of HIV infected older adolescents and youth (15–24 years) enrolling into care over a six-year period compared to younger adolescents [13]. Another study following up adolescents and youth in 4 sub-Saharan countries documented a 6% increase in the number of youth aged 15–24 years enrolling into HIV care (from 12% in 2005 to 18% in 2010) [14]. These findings can be attributed to the increase in HIV incidence that has been noted in this age group over the study period [15], as a result of underlying increased vulnerability to HIV infection, targeted HIV testing for youth and young adults [16–18].

On-site VCT services contributed the largest proportion of new pre-ART enrolees in our cohort. For large busy hospitals, on-site VCT services offer excellent opportunities for people to access HIV testing services. Upward trends in the proportion of enrolments from the VCT were also documented in a prospective cohort analysis of adult patients in Tanzania [19], and in a multi-country retrospective data review of older HIV positive adults [20]. A recent retrospective study in Ethiopia however, reported an increase in the proportion of adults enrolling into care through PITC services over time [21]. However, this analysis was done at a time when PITC services were being scaled up in the country which may have biased the findings. PITC has been shown to have a higher yield in identifying later stage HIV positive patients [22].

Overall, about 45% of patients were linked to HIV care within the same year of diagnosis. Although this falls well below the 80% national average linked within 3 months of diagnosis in 2012 [23], it is consistent with proportions documented from studies in other SSA settings, which range from 38% [22] to 68% [24] who are linked to care within six months of diagnosis.

We noted an increase in the proportion of patients initiating care in early disease (WHO stage I & II), and with higher CD4 cell counts. Similar temporal trends have been observed in several SSA countries in the last decade [19, 25–28]. This is a positive program indicator. It is expected that early entry into care will translate into prompt initiation of ART prior to severe immune damage [29].

Male gender, the youth (20–24 years), adults aged 25–34 years, inadequate social support (defined as single, or divorced marital status), urban residency, low CD4 cell counts, and CD cell counts above 500 cells/µl were all associated with increased risk of loss to follow-up from pre-ART care.

Our analysis found patients with CD4 counts ≤100 cells/µl to be at higher risk of attrition. This is in contrast to other studies which reported higher CD4 counts (>250 cells/µl) to be predictive of attrition [30, 31]. Our definition of attrition included both mortality and loss to follow up while the other studies referenced specifically focused on loss to follow up, hence the different findings. Indeed, patients with advanced immunosuppression are at increased risk of death [32]. These findings underscore the importance of initiating ART at higher CD4 counts. Our finding of CD4 counts >500 to be predictive of attrition is consistent with that of other studies [30, 31].

Previous work has documented younger age (<35 years) to be predictive of pre-ART loss to follow up [30, 33–35]. Similarly, our analysis found evidence of higher attrition in the 20–24 and 25–34 years age groups.

Consistent with other findings, being single or divorced was predictive of attrition [34–36]. This possibly implies limited social and financial support mechanisms. Implementing strategies that address social and economic needs could help to mitigate attrition, especially among socially vulnerable patients.

The strengths of this study include the reasonably large sample size which rendered sufficient power for precise effect estimates, and the long duration of follow up which allowed for trends analyses over time. Study limitations are inherent in the data source. We used routinely collected clinical data, which is more prone to errors, including missing information [21].

## Conclusions

We have documented an upward trend in numbers of adolescents and youth entering care. Upstream, this may point to increasing new infections in this age category; and therefore the need to re-examine HIV prevention interventions to better target adolescent and young people’s needs. Down-stream, HIV care and treatment programs may need to better prepare to deliver care to adolescents and youth. Overall, the proportion of patients enrolling in care with early HIV increased over time. This is expected to translate into better treatment outcomes and the need for less intense monitoring at the initiation of ART, thereby releasing valuable resources to the few vulnerable patients at risk of early mortality and severe disease.

## Abbreviations

ART: antiretroviral therapy
SSA: Sub-Saharan Africa
WHO: World Health Organisation
VCT: Voluntary Counselling and Testing Centre
KNH CCC: Kenyatta National Hospital Comprehensive Care Centre
PEPFAR: President’s Emergency Plan for AIDS Relief
PITC: provider initiated testing and counselling
TB: tuberculosis
EHR: electronic health records
